# Inhibition of Proliferation, Migration and Proteolysis Contribute to Corticosterone-Mediated Inhibition of Angiogenesis

**DOI:** 10.1371/journal.pone.0046625

**Published:** 2012-10-02

**Authors:** Eric A. Shikatani, Anastassia Trifonova, Erin R. Mandel, Sammy T. K. Liu, Emilie Roudier, Anna Krylova, Andrei Szigiato, Jacqueline Beaudry, Michael C. Riddell, Tara L. Haas

**Affiliations:** 1 School of Kinesiology and Health Science, York University, Toronto, Ontario, Canada; 2 Department of Biology, York University, Toronto, Ontario, Canada; 3 Muscle Health Research Centre, York University, Toronto, Ontario, Canada; Ottawa Hospital Research Institute, Canada

## Abstract

The angiostatic nature of pharmacological doses of glucocorticoid steroids is well known. However, the consequences of pathophysiological elevation of endogenous glucocorticoids are not well established. In the current study, we hypothesized that the angiostatic effect of corticosterone, an endogenous glucocorticoid in rodents, occurs through multi-faceted alterations in skeletal muscle microvascular endothelial cell proliferation, migration, and proteolysis. Chronic corticosterone treatment significantly reduced the capillary to fiber ratio in the tibialis anterior muscle compared to that of placebo-treated rats. Corticosterone inhibited endothelial cell sprouting from capillary segments *ex vivo*. Similarly, 3-dimensional endothelial cell spheroids treated with corticosterone for 48 hours showed evidence of sprout regression and reduced sprout length. Endothelial cell proliferation was reduced in corticosterone treated cells, coinciding with elevated FoxO1 and reduced VEGF production. Corticosterone treated endothelial cells exhibited reduced migration, which correlated with a reduction in RhoA activity. Furthermore, corticosterone treated endothelial cells in both 3-dimensional and monolayer cultures had decreased MMP-2 production and activation resulting in decreased proteolysis by endothelial cells, limiting their angiogenic potential. Promoter assays revealed that corticosterone treatment transcriptionally repressed MMP-2, which may map to a predicted GRE between −1510 and −1386 bp of the MMP-2 promoter. Additionally, Sp1, a known transcriptional activator of MMP-2 was decreased following corticosterone treatment. This study provides new insights into the mechanisms by which pathophysiological levels of endogenous glucocorticoids may exert angiostatic effects.

## Introduction

In order to increase the vascular supply to a tissue, new capillaries must grow from the existing vasculature in a process known as angiogenesis [1], [2], [3]. Early studies by Folkman et al. [4] found that pharmacological (millimolar) concentrations of the glucocorticoid steroid hydrocortisone prevented heparin-stimulated angiogenesis in the chick chorioallantoic membrane and induced the regression of capillaries within the surrounding tissue. These angiostatic effects were also observed with synthetic glucocorticoids such as dexamethasone and epicortisol [5]. Notably, Small et al. [6] showed that the endogenously produced glucocorticoid steroid corticosterone administered in pathophysiological doses (300–600 nM) could inhibit angiogenesis *in vitro* and *in vivo*, providing the first evidence for the angiostatic role of endogenous corticosteroids. More recently, it has been reported that increased endogenous glucocorticoids due to aging and pharmacologic hyperglucocorticoidism results in decreased bone angiogenesis, possibly by downregulating hypoxia-inducible factor-1α transcription and production of vascular endothelial growth factor (VEGF) by osteoblasts and osteocytes [7]. Logie et al. [8] showed that cortisol, the main endogenous glucocorticoid in humans, could inhibit the organization of human umbilical vein endothelial cells into tube-like structures. They proposed that the mechanism may involve cytoskeletal changes of endothelial cells and increases in anti-angiogenic factors such as thrombospondin-1. However, little is known about the cellular signaling through which endogenous glucocorticoids exert their effects.

Angiogenesis occurs through a series of events that include endothelial cell proliferation and the invasion and migration of sprouting cells through the interstitial matrix. VEGF is a potent pro-angiogenic molecule that stimulates and maintains endothelial cell proliferation and cell motility during sprouting angiogenesis [9], [10]. Cytoskeletal rearrangement and cellular morphology play a large part in endothelial cell sprouting and migration. Förster et al. [11] reported that prolonged treatment of brain endothelial cells with dexamethasone caused the formation of cortical actin rings and increased cell-cell junction formation. It has also been reported that glucocorticoids can inhibit cell migration by increasing the formation of thick stress fibers and focal adhesions [12]. The RhoGTPases have established roles in cell motility, cytoskeletal reorganization and junction formation [13], [14], [15]; thus, RhoGTPase-mediated changes to cell morphology and cytoskeletal rearrangement with corticosterone treatment may contribute to the angiostatic effects of glucocorticoid steroids.

Proteolysis of the capillary basement membrane and interstitial matrix enables the sprouting and migration of endothelial cells. Matrix degradation is mediated by matrix metalloproteinases (MMPs), a family of proteases that cleave extracellular matrix (ECM) proteins, growth factors and cell surface receptors, and are required for the process of angiogenesis [16], [17], [18]. MMP-2 is secreted by endothelial cells and is crucial for angiogenesis to occur [19], [20]. Activation of secreted latent pro-MMP-2 occurs on the cell surface through the formation of a ternary complex of membrane type 1-MMP (MT1-MMP), tissue inhibitor of metalloproteinase-2 (TIMP-2) and MMP-2 [21], [22]. Excess TIMP-2 can occupy all available binding sites for MT1-MMP, preventing cleavage of pro-MMP-2. Glucocorticoid regulation of MMP-2 production and activity in microvascular endothelial cells has not been reported, although a negative correlation between plasma cortisol and MMP-2 levels was observed in humans subjected to a stress stimulus [23] or following direct injection of hydrocortisone [24]. Such regulation may be transcriptionally mediated, as promoter regions of other MMPs such as MMP-1 are known to contain a glucocorticoid response element (GRE) [25]. Additionally, glucocorticoids have been shown to induce mRNA production of TIMP-1 and 2 [11], [26], which would also contribute to inhibition of proteolysis.

The cellular mechanisms by which pathophysiological levels of glucocorticoids inhibit angiogenesis remain incompletely understood. It was hypothesized that impaired proliferation and migration, together with reduced production of MMPs by endothelial cells contribute to corticosterone-mediated inhibition of angiogenesis.

**Figure 1:**
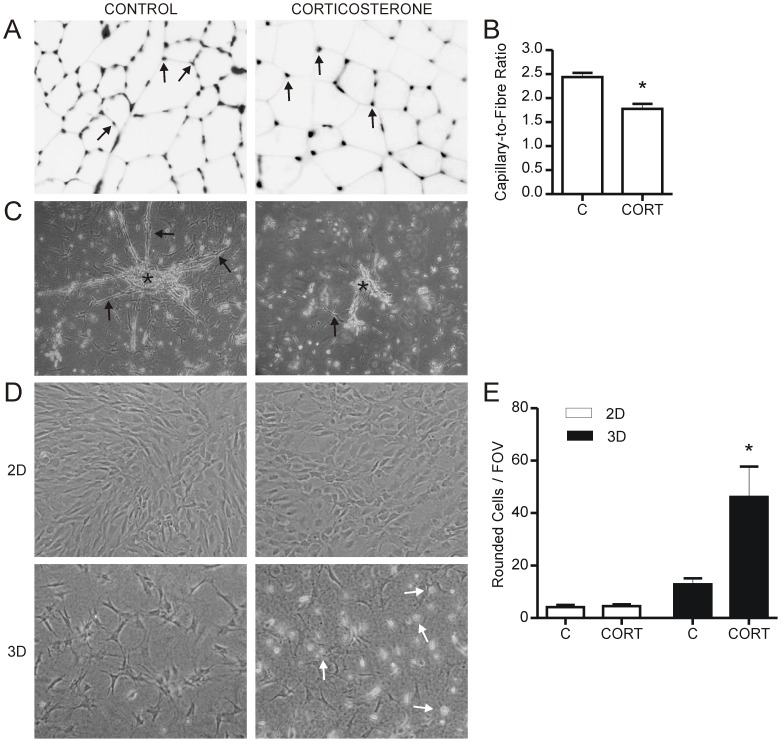
Corticosterone inhibits angiogenesis *in vivo* and *ex vivo*. Tibialis anterior muscles from rats implanted with either wax (Control) or corticosterone pellets (n = 5 per group) were sectioned and stained for capillaries using Griffonia Simplicifolia isolectin-fluorescein. Images have been inverted to enhance visualization of the muscle fibers. Arrows indicate capillaries (A). Capillary to fiber counts were made on 5 representative fields of view per rat (*p = 0.002, vs. control) (B). Isolated capillary segments were embedded in type I collagen and treated with 600 nM corticosterone for 72 hours. Capillary segments are indicated by an asterisk (*), while arrows indicate sprouts arising from the original segment (51× magnification) (C). Skeletal muscle endothelial cells suspended in a 3-dimensional type I collagen culture treated with corticosterone displayed increased rounding of cells (indicated with white arrows), which was not evident in monolayer (2D) cultures (D, E *p = 0.04 vs 3D Control). C – Control, CORT – Corticosterone.

## Materials and Methods

All experiments were approved by the York University Animal Care Committee in accordance with the Canadian Council for Animal Care guidelines (approval #2010-15(R1) and #2010-28(R3)).

### Rat Treatment with Corticosterone

Sprague-Dawley rats (age 6 weeks, 200–250 g; N = 10) from Charles River Laboratories (Montreal, QC, Canada) were implanted either with four wax placebo pellets (n = 5), or with pure corticosterone pellets (n = 5; 100 mg/pellet, Sigma, Cat # C2505), placed subcutaneously between their scapulae under aseptic conditions, as previously described [27]. Plasma was collected six days following surgery via tail vein lancet (∼20 µl) using a sterile scalpel at 0900 h and at 2000 h to measure morning basal (fed) and peak evening corticosterone levels using a commercially available kit (MP Biomedicals, Solon, OH, Cat# 07-120102). This procedure typically elevates basal corticosterone levels (∼50 ng/ml) to ∼500 ng/ml, but does not change peak corticosterone levels in Sprague-Dawley rats [27]. Animals were provided food and water *ad libitum* for 2 weeks prior to sacrifice and removal of muscle for analysis. After 14 days, animals were euthanized and the tibialis anterior was frozen for histological analysis. Muscle cross-sections were stained for capillaries using Griffonia Simplicifolia isolectin-fluorescein (Vector Labs). Capillary to fiber counts were performed independently by a blinded observer, averaging the values of 5 representative fields of view per rat.

### Capillary Sprout Formation Assay

Capillary fragments were isolated from Sprague-Dawley rat epididymal fat pad and cultured within a three-dimensional (3D) type I collagen matrix (Vitrogen) as previously described [28]. Capillary segments were treated with 600 nM corticosterone (Tocris #3685) for 72 hours. After 48 hours, sprouts were photographed using a Canon Powershot G5 digital camera mounted to a Zeiss Axiovert 40C microscope.

**Figure 2:**
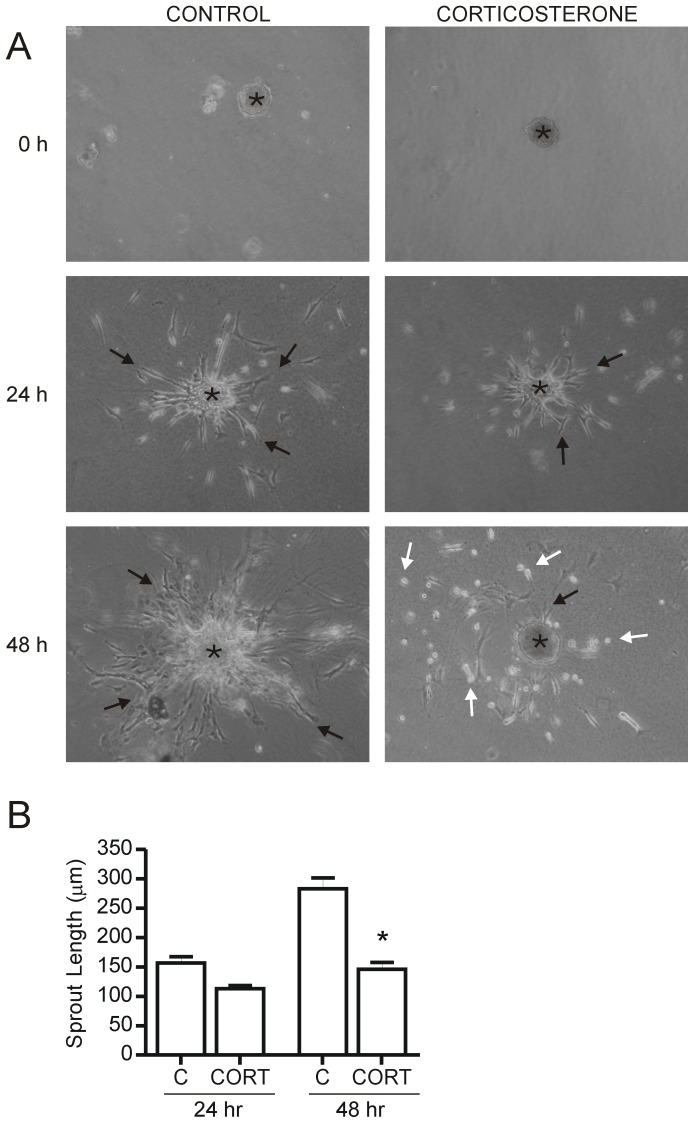
Corticosterone destabilizes newly formed endothelial cell sprouts. Skeletal muscle endothelial cell spheroids were suspended in a 3-dimensional type I collagen culture. Spheroids were treated for 24 and 48 hours with 600 nM corticosterone. Spheroids are indicated by an asterisk (*), sprouting cells are indicated by black arrows, and rounded cells are indicated by white arrows (51× magnification) (A). 48 hours of corticosterone treatment significantly decreased sprout length compared to control spheroid sprout lengths (*p<0.05 vs 48 hr control, n = 28–35 sprout lengths from 5 independent experiments) (B). C – Control, CORT – Corticosterone.

### Cell Culture

All *in vitro* experiments were performed with skeletal muscle microvascular endothelial cells isolated from Sprague-Dawley rat extensor digitorum longus muscles cultured in gelatin coated flasks, as described previously [29]. Cells used were from between passages 4 to 9.

The protocol for culturing human umbilical vein endothelial cell spheroids [30] was adapted for use with rat skeletal muscle microvascular endothelial cells. Endothelial cells were plated at a density of 1000 cells/well in 96-well round bottom plates in 50 µl 0.8% medium viscosity carboxymethylcellulose (Sigma-Aldrich #C4888) in complete DMEM. Spheroids were formed by incubating 96-well plates at 37°C, 7% CO_2_ for 4 to 7 days. Spheroids were lifted by brief trypsinization and then resuspended in neutralized acid-solubilized 2.5 mg/ml rat tail type 1 collagen (BD Biosciences #354236). Collagen-embedded spheroids were treated with corticosterone for 24 and 48 hours. Digital images of the spheroids were captured and sprout lengths quantified using Metamorph image analysis software.

For monolayer cultures, 1.0×10^6^ cells were plated on type 1 collagen-coated 35 mm^2^ dishes and treated with corticosterone for 24 and 48 hours. In some experiments, cells were pretreated with GR inhibitor RU 486 (10 µM, Tocris #1479) 2 hours prior to corticosterone treatment. Triton lysis buffer (120 mM Tris, 0.1% Triton X-100, 5% glycerol) supplemented with 10% protease inhibitor cocktail (Sigma #P8340) and 1.1 µM Na_3_VO_4_ was used in the preparation of whole cell lysates. Protein concentrations were determined using a bicinchonic acid assay (BCA, Pierce), as per manufacturer’s instructions. Alternatively, cells were lysed using Cells-to-cDNA™ lysis buffer (Ambion) for RNA analysis.

For 3D cultures, cells were resuspended in type 1 collagen at a density of 5.0×10^5^ cells/ml, as previously described [31]. Cells were treated with corticosterone for 24 and 48 hours. Media was collected and collagen droplets were minced with a scalpel and were then lysed with Triton lysis buffer.

### Microscopy

Monolayer and 3D cultures of endothelial cells or endothelial cell spheroid cultures were visualized using a Zeiss Axiovert 40C microscope (10X objective) and images were captured by digital camera (Canon Powershot G5). Cultures were fixed with 4% paraformaldehyde, permeabilized and immunostained with anti-cleaved caspase-3 (1∶500; Cell Signaling #9664), and counterstained with FITC-phalloidin (Sigma #P5282) to visualize actin filaments. Staining was visualized using a Zeiss Axiovert 200 M microscope and images were captured and analyzed using Metamorph software (Universal Imaging). In some cases, confocal microscopy was utilized to image the actin cytoskeleton (Olympus Fluoview 300, Argon laser, 488 nm, x40 objective, pinhole aperture 2). 2D and 3D cultured cells were treated with corticosterone for 48 hours, and morphology was documented using the Axiovert 40C as described above. Cell death was assessed using the LIVE/DEAD cell imaging kit (Invitrogen #R37601), according to manufacturer’s directions. Stained cells were imaged using the Axiovert 200 M, as described above.

**Figure 3:**
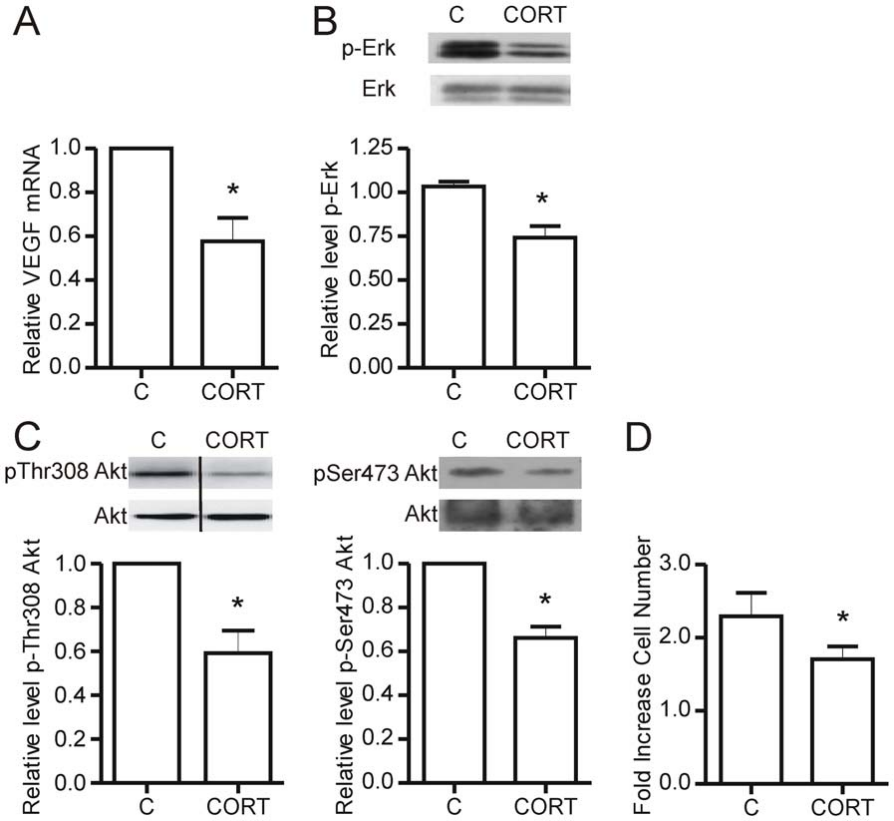
Corticosterone reduces VEGF and inhibits endothelial cell proliferation. Endothelial cells (1.0×10^6^ cells) were plated in 35 mm^2^ dishes coated with type I collagen and treated with 600 nM corticosterone for 48 hours. Whole cell lysates were used for qRT-PCR or Western blotting. VEGF mRNA levels were decreased with corticosterone treatment (*p = 0.01 vs conrol, n = 6) (A). ERK1/2 (*p = 0.01 vs control, n = 5) (B) and Akt phosphorylation were decreased with corticosterone treatment (pThr308: *p = 0.03 vs control, n = 4; pSer473: *p = 0.003 vs control, n = 5) (C). Representative immunoblots are shown. Endothelial cell proliferation was determined using a CyQuant Cell proliferation kit (Molecular Probes). After 48 hours, endothelial cell proliferation was decreased with corticosterone treatment (*p = 0.02 vs. control, n = 5) (D). C – Control, CORT – Corticosterone. N values represent independent experiments.

### Quantitative RT-PCR

Lysates were DNase treated, and then RNA was reverse-transcribed using MMLV reverse transcriptase (New England Biolabs). cDNA samples were analyzed by qPCR using PCR supermix (Invitrogen Canada; Burlington, ON, Canada P/N 11743) and Taqman probes (Applied Biosystems) for rat GAPDH (Rn99999916_s1), rat FoxO1 (Rn01494868_m1), rat MMP-2 (Rn02532334_s1) and VEGF (Rn00582935_m1). Real-time PCR analysis was conducted using the ABI PRISM 7700 Sequence Detector System (Applied Biosystems). The comparative Ct method was used to determine relative quantification of mRNA expression, using GAPDH as the housekeeping gene.

### Endothelial Cell Proliferation Assay

The CyQUANT Cell Proliferation assay kit (Invitrogen #C7026) was utilized to assess cell proliferation. Cells were plated in duplicate 96 well plates, in serial dilutions from 50 to 50000 cells. Corticosterone (600 nM) was added to 1 set of duplicate plates. After an attachment period of 4 hours, media was removed from one of the microplates ( = T_4_), and the plate was frozen until further analysis. 48 hours after plating, this procedure was repeated for 2^nd^ microplate ( = T_48_). All samples were thawed, incubated with an appropriate dilution of CyQUANT GR dye in lysis buffer, then fluorescence was measured with a microplate reader (Wallac) equipped with 480 nm excitation and 520 nm emission filters. Proliferation values (T_48_) were calculated as an increase in intensity above T_4_. Results from four independent experiments were analyzed.

### Scrape Migration Assay

For migration assays, 2.0×10^6^ cells were plated on 60 mm^2^ dishes and pre-treated with corticosterone for 48 hours. Cells were then treated with Mitomycin C (5 µg/ml, Sigma #M4287) to inhibit cell proliferation for 2 hours before a scrape was created in the monolayer using a cell scraper. Dishes were marked with a grid to allow cell position to be determined at various time points. Cells were observed under bright field microscopy, and images captured at 0 and 24 hours after scrape. Total distance migrated by cells in 24 hours was determined as the difference of the cell front relative to the 0 hour timepoint. Migration distance was measured using Metamorph software. Three to four independent fields of views were captured, with three distance measurements per field of view.

### RhoGTPase Activity Assay

For RhoGTPase activity assays, 2.0×10^6^ cells were plated on 100 mm^2^ dishes, treated with corticosterone for 48 hours, and then trypsinized and re-plated on 100 mm^2^ dishes coated with type 1 collagen at a density of 1.0×10^6^ cells/dish. Corticosterone treatment was continued during adhesion. After two hours, adherent cells were lysed (50 mM Tris, 10 mM MgCl_2_, 0.3 M NaCl, 2% NP-40 supplemented with 10% protease inhibitor cocktail, 1 mM Na_3_VO_4_, 1 mM NaF, and 1 mM PMSF). Cell lysates were incubated on a rotisserie at 4°C for 2 hours with agarose beads coated with either Rhotekin binding domain (Cytoskeleton #RT02) or Rac/Cdc42 (p21) activated kinase 1 (PAK) binding domain (Cytoskeleton #PAK02). Beads were washed three times in IP wash buffer (50 mM Tris, 10 mM MgCl_2_, 0.3 M NaCl, 2% NP-40), and prepared in 60 µl denaturing loading buffer and assessed using Western blotting.

### Gelatin Zymography

5 µg (3D) or 10 µg (2D) of protein from cell lysates or 4.5 µl media (3D) were prepared in a non-denaturing loading buffer and separated through an 8% SDS-polyacrylamide gel containing 0.08% gelatin, as described previously [28]. Densitometric analysis was done using FluorChem software (AlphaInnotech). Total MMP-2, indicative of MMP-2 production, was analyzed as the sum of the latent (72 kDa) and activated (62 kDa) MMP-2 bands. Percent activated MMP-2, often an indirect indicator of the amount of MT1-MMP, is calculated as the ratio of activated MMP-2 to total MMP-2 [28].

### Western Blotting

Samples were prepared in denaturing loading buffer, separated through SDS-polyacrylamide gels (10 or 15%) and then transferred to an Immobilon-P PVDF membrane (Millipore) using a semi-dry transfer method. Protein detection was conducted using primary antibodies against phosphorylated c-jun (Ser63), phospho-Erk1/2 (Thr202/Tyr204), FoxO1, Rac 1/2/3, RhoA or Cdc42 (all at 1∶1000, Cell Signaling #9261; 9101; 2880; 2467; 2117; 2462, respectively), phospho-Akt (Thr308 or Ser473), Akt (all at 1∶500, Cell Signaling #2918; 4058; 9272, respectively), MT1-MMP (1∶1000, Novus #110-57216), TIMP-2 (1∶500, Chemicon #AB8107), Sp1 (1∶250, Santa Cruz #sc-59), GATA-2 (1∶500, Santa Cruz #sc-9008), followed by anti-rabbit IgG Horseradish Peroxidase (1∶10000, GE Biosciences #NA931V). Detection was performed using enhanced chemiluminescent reagents (Millipore #34080 or Pierce # WBKLS0100) and exposure to CL-XPosure film (Thermo Scientific #34090) or detection by digital imaging system (Kodak MM4000Pro). Membranes were stripped and reprobed with antibodies against the control protein α/β-tubulin, β_1_-integrin or β-actin (all at 1∶1000, Cell Signaling #2148; 4706; 4967, respectively), to normalize for loading. Densitometric analysis was performed on films using FluorChem software (AlphaInnotech). For quantification, bands were first normalized to loading controls and then normalized to their respective experimental controls.

### Surface Biotinylation

Monolayer cultures were treated for 48 hours with corticosterone. Prior to lysing, cells were incubated with 1 ml of Ez-link Sulfo-NHS-biotin (1 mg/ml, Pierce #21217) for one hour on ice. Biotinylation was quenched with 100 mM glycine for 20 minutes, and then cells were lysed. 100 µl of streptavidin-agarose resin (Thermo Scientific #20347) was added to 75 µg of sample protein, filled to a volume of 500 µl with 1% NP-40, and rotated at 4°C for two hours. Agarose resin beads were pelleted and resuspended in 60 µl denaturing loading buffer and analyzed by Western blotting.

### Secreted TIMP- 2

Monolayer cultures were treated for 48 hours with corticosterone. For the last 4 hours of treatment, media was replaced with OptiMEM serum free media (Invitrogen) and collected at the end of the treatment period, and then cells were lysed as previously described. OptiMEM was concentrated in centrifugal filters with a 10 kDa cut off (Millipore #UFC801024), and then protein concentration was quantified as previously described. Media and lysates were analyzed using Western blotting as previously described.

**Figure 4:**
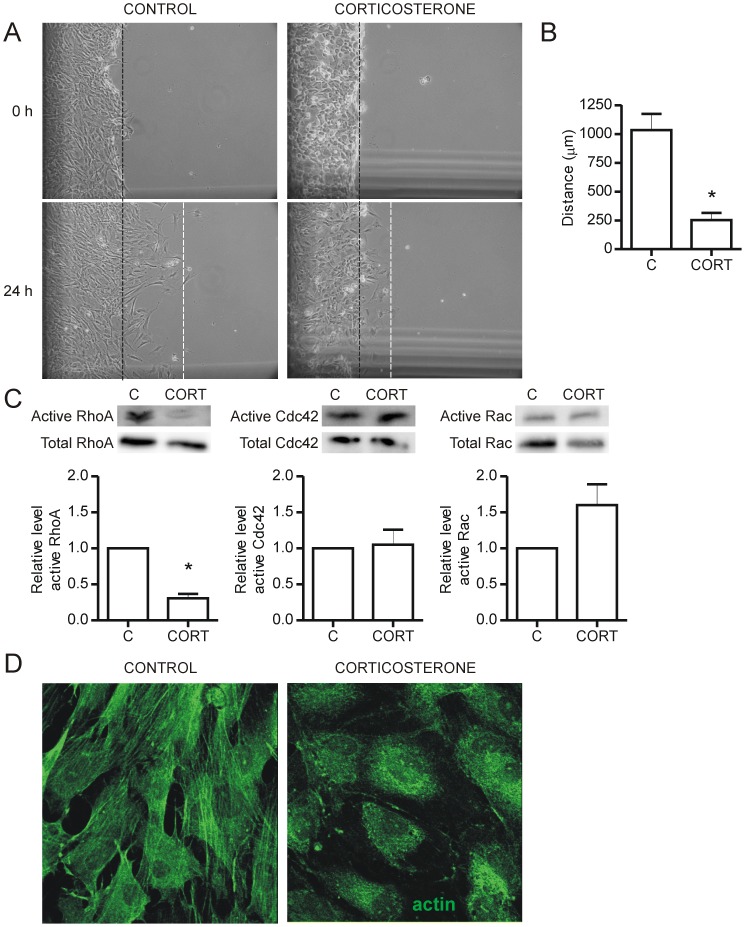
Corticosterone inhibits endothelial cell migration. Endothelial cells (2.0×10^6^) were plated in 60 mm^2^ plates coated with 1.5% gelatin and treated with 600 nM corticosterone for 48 hours. Cells were pretreated with 5 µg/ml Mitomycin C for 2 hours prior to removing cells from half of the dish using a rubber cell scraper. Cells then were allowed to migrate into the scrape area for 24 hours (A). The dashed black line denotes the edge of the scrape at time 0, and the dashed white line denotes migration front after 24 hr. Migration distance was calculated as the difference between white and black lines in each field of view. Corticosterone inhibited migration of endothelial cells into the scrape area (*p<0.0001 vs. control, n = 11 measurements from 3–4 fields of view) (B). RhoGTPase activity assays were performed using PAK or Rhotekin coated beads on endothelial cells pre-treated with 600 nM corticosterone for 48 hours then plated on type I collagen for 2 hr prior to lysis. Western blots were performed for RhoA, Cdc42 and Rac1 (*p = 0.008 vs. control, n = 3 independent experiments) (C). Corticosterone treated cells were stained with FITC-Phalloidin to visualize actin. Stress fibers were not observed with corticosterone treatment (D). C – Control, CORT – Corticosterone.

### MMP-2 Promoter Activity Assay

Rat MMP-2 promoter-luciferase constructs (−1686 bp, −1560 bp, −1368 bp and −510 bp in pGL3) [29] and control Renilla luciferase were transiently transfected into endothelial cells using Lipofectamine LTX and Plus reagent (Invitrogen), according to the manufacturer’s instructions. Following transfection, cells were treated with corticosterone for 48 hours. Firefly and Renilla luciferase activities were measured using a dual-luciferase activity kit (#E2920, Promega), as per manufacturer’s instructions (using a Wallac plate reader). All values for conditions were calculated as a ratio to the empty vector condition and normalized according to Renilla values to account for well-to-well variations in transfection efficiency.

### Adenoviral Transduction of FoxO1

Adenoviral transduction of primary microvascular endothelial cells using Adeno-CAFoxO1 (100 pfu per cell, generously provided by Dr.Robert Gerard, University of Texas Southwestern) was performed to over-express constitutively active FoxO1. Adeno-βgal transduction was used as a negative control. 24 hours following transduction, cells were lysed for subsequent analysis of protein (RIPA lysis buffer) and mRNA (Cells to cDNA lysis buffer; Ambion).

**Figure 5:**
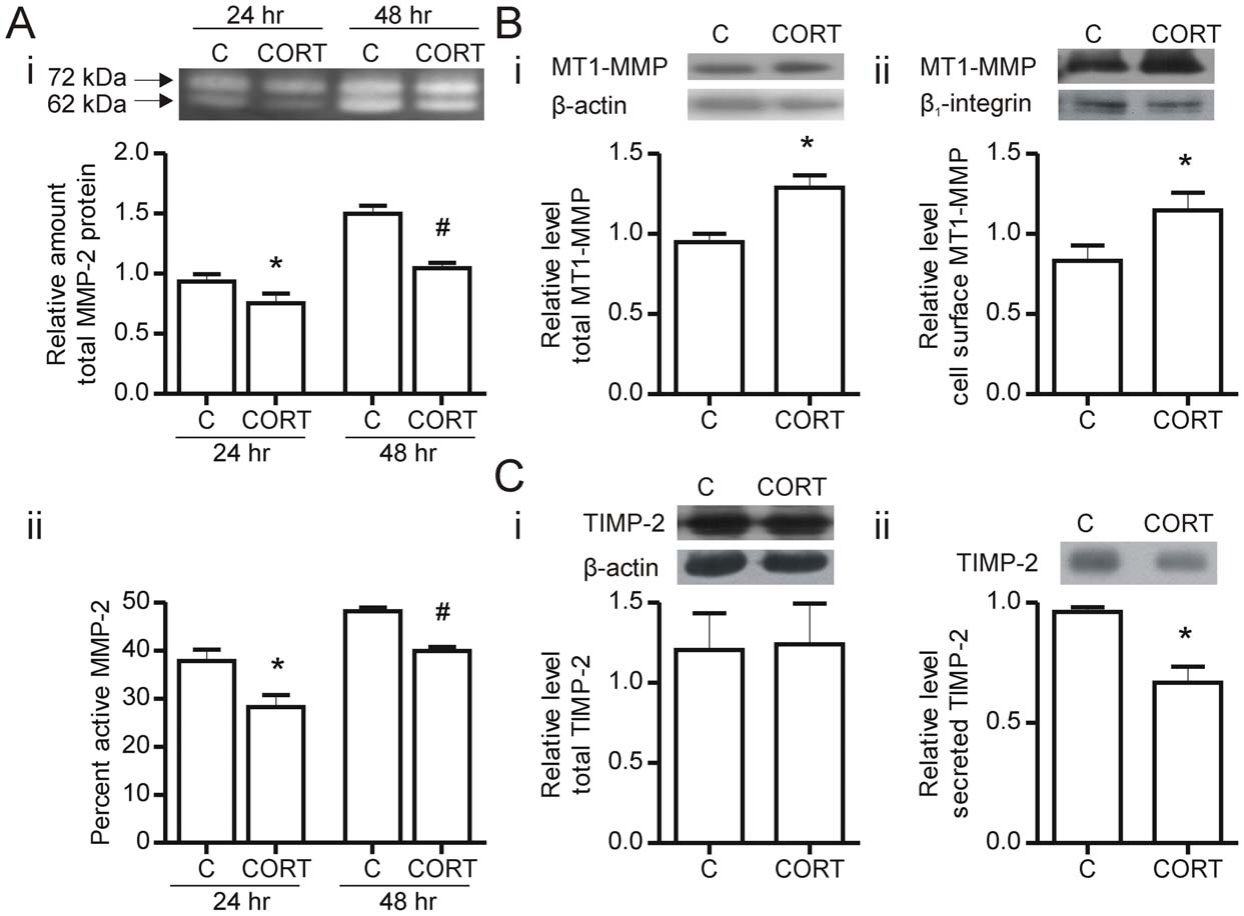
Corticosterone inhibits MMP-2 production and activation. Endothelial cells were resuspended in type 1 collagen at a density of 5.0×10^5^ cells/ml, and treated with 600 nM corticosterone for 24 and 48 hours. Total (Ai) and percent active (Aii) MMP-2 levels in cell culture media were measured by gelatin zymography; (i) * p = 0.0446 vs 24 hr control, # p = 0.012 vs 48 hr control. (ii) * p = 0.0001 vs 24 hr control, # p = 0.008 vs 48 hr control, n = 4, respectively. (A). Total MT1-MMP was detected by immunoblotting of whole cell lysates (Bi) while cell surface MT1-MMP was detected by immunoblotting of cell surface biotinylated proteins (Bii) (*p = 0.006 vs control, n = 4; *p = 0.03 vs control, n = 4, respectively). Whole cell TIMP-2 levels (Ci) and secreted TIMP-2 (Cii) were detected by immunoblotting (*p<0.05 vs control, n = 3). C – Control, CORT – Corticosterone. N values represent independent experiments.

### Statistical Analysis

One-way analysis of variance (ANOVA) followed by Tukey post-hoc tests or Student’s t-tests were performed to determine statistical significance (p<0.05) using GraphPad Prism 5. All cultured cell experiments were repeated independently a minimum of 3 times. Data are presented as mean ± standard error of the mean.

## Results

### Chronic Corticosterone Treatment Reduces Capillary Number in Rat Skeletal Muscle and Inhibits Capillary Sprouting *in vitro*

Subcutaneous implantation of Sprague-Dawley rats with corticosterone pellets resulted in elevated basal plasma corticosterone levels (42±14 vs 508±67 ng/ml in the control and corticosterone treated groups respectively) but unchanged peak plasma corticosterone levels (451±82 vs 598±107 ng/ml), consistent with previous observations [27]. Capillary to fiber ratio in the tibialis anterior muscle was reduced by approximately 30% in rats that received chronic treatment of corticosterone compared to control rats (Control, 2.44±0.09 vs. Corticosterone, 1.78±0.10; p = 0.002) (**Figure 1A, B**). This could be indicative either of regression (rarefaction) of pre-existing capillaries or the inhibition of new capillary growth. To more specifically examine the cellular effects of corticosterone on endothelial cells, we turned to several *in* *vitro* models. Capillary segments were embedded within a 3D collagen matrix and then treated with 600 nM corticosterone for 48 hrs, which resulted in decreased endothelial cell sprouting compared to control (**Figure 1C**). When monolayer (2D) and 3D type I collagen cultures (3D) of endothelial cells were treated with corticosterone, phenotypic differences were apparent only in 3D cultured endothelial cells, which exhibited an increased number of rounded cells (**Figure 1D, E**).

**Figure 6:**
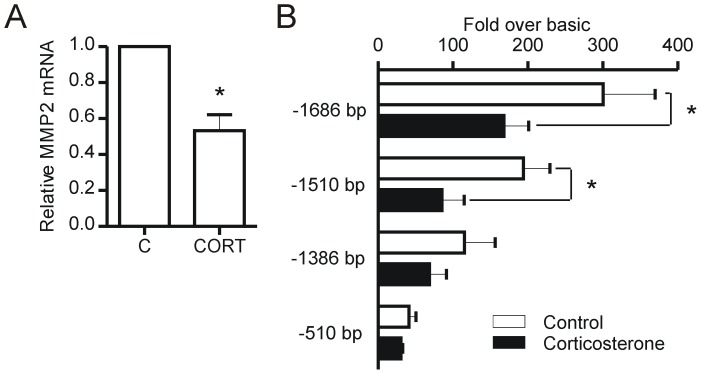
Corticosterone treatment decreases MMP-2 promoter activity. Endothelial cells (1.0×10^6^ cells) were plated in 35 mm^2^ dishes coated with type I collagen and treated with 600 nM corticosterone for 48 hours. Whole cell lysates were used for qRT-PCR. MMP-2 mRNA levels were decreased with corticosterone treatment (A) (p = 0.006, n = 5). Endothelial cells (7.5×10^5^ cells) were plated in 35 mm^2^ dishes coated with type I collagen for 24 hours before being transfected with plasmid DNA encoding full length MMP-2 promoter (−1686 bp), or truncated promoter (−1510 bp, −1386 bp, −510 bp) coupled to firefly luciferase (pGL3basic) and then treated with 600 nM corticosterone for 48 hours (B). Renilla luciferase (pRL) was transfected into each well as a control to normalize transfection efficiency. Relative light units were calculated as a ratio to the empty vector condition (pGL3basic) and normalized according to Renilla values to account for well to well variations in transfection efficiency (*p<0.05 vs control, n = 4 independent experiments).

To further assess the effect of corticosterone on sprouting, we utilized an endothelial cell spheroid assay in which endothelial cells migrate radially from a central cell cluster. After 24 hours of culture, corticosterone treated spheroids did not appear markedly different from control spheroids as both displayed substantial sprouting and migration of endothelial cells from the surface of cell spheroids (Control, 157±11 µm vs. Corticosterone, 113±5 µm, p>0.05) (**Figure 2A**). After 48 hours, sprouts in control cultures had extended significantly further than at the 24 hour time point, while sprouts in the corticosterone treated condition appeared to have stopped migration (Control, 283±18 µm vs. Corticosterone, 146±12 µm; p<0.05) (**Figure 2B**). In the corticosterone treated cultures, cells frequently appeared rounded and some sprouts were fragmented. Staining for markers of apoptosis and necrosis in 2D, 3D and spheroid cultures revealed that corticosterone treated cells were not apoptotic or necrotic (**Figure S1**).

**Figure 7:**
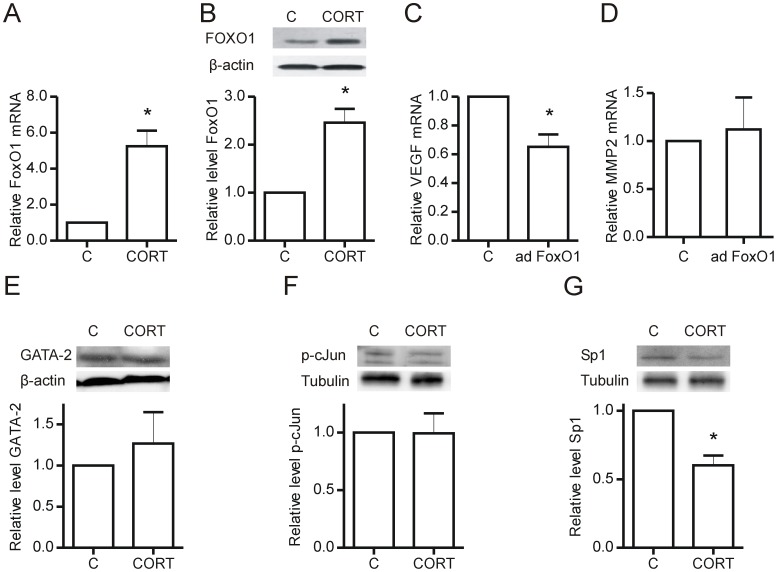
Corticosterone regulates FoxO1 and Sp1. Endothelial cells (1.0×10^6^ cells) were plated in 35 mm^2^ dishes coated with type I collagen and treated with 600 nM corticosterone for 48 hours. Whole cell lysates were used for qRT-PCR or Western blotting. FoxO1 mRNA (A) and protein (B) increased significantly with corticosterone treatment (*p = 0.04 vs control; n = 3 and *p = 0.01 vs control, n = 4, respectively). Adenoviral overexpression of FoxO1 resulted in decreased VEGF mRNA (*p = 0.03 vs control, n = 4) (C), but did not change MMP-2 mRNA levels (n = 4) (D). GATA-2 (E), phosphorylated c-Jun (F) and Sp1 (G) levels were determined by Western blotting of corticosterone treated whole cell lysates, normalized either to tubulin or β-actin (*p = 0.03 vs control, n = 3 independent experiments). C – Control, CORT – Corticosterone, ad FoxO1– Adenovirally transduced constitutively active FoxO1.

### Corticosterone Reduces VEGF mRNA and Inhibits Endothelial Cell Proliferation

Corticosterone treated endothelial cells had reduced VEGF mRNA levels compared to control cells (p = 0.01) (**Figure 3A**). VEGF promotes cell proliferation and migration through multiple signal pathways. Consistent with the reduced VEGF production, cells also exhibited decreased phosphorylation of ERK1/2 (p = 0.006) and Akt (Thr308: p = 0.03; Ser473: p = 0.02) following 48 hours of corticosterone treatment (**Figure 3B, C**). Furthermore, the proliferation rate of corticosterone treated endothelial cells was decreased compared to control cells (p = 0.02) (**Figure 3D**).

**Figure 8:**
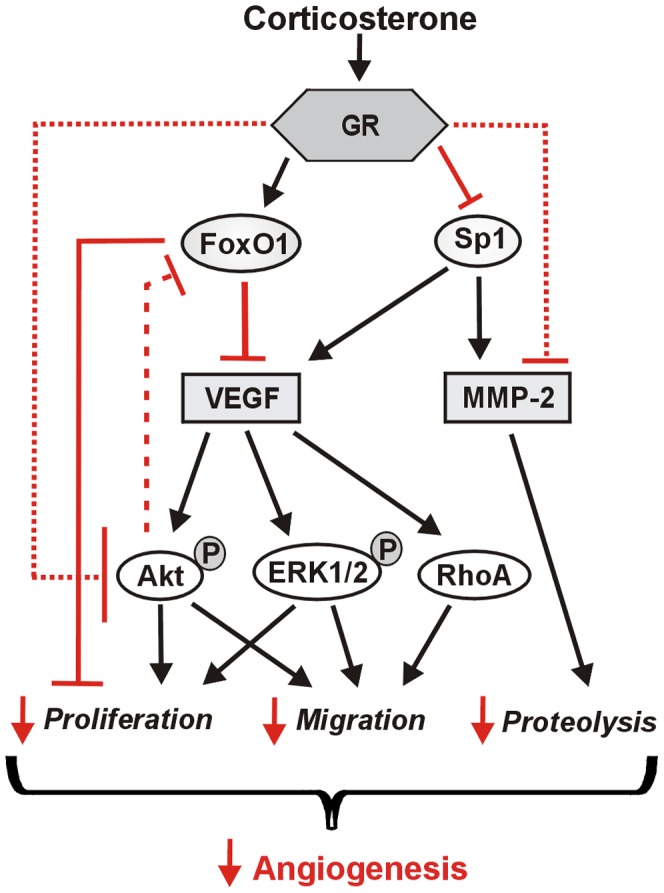
Corticosterone inhibits multiple pathways to repress angiogenesis. Corticosterone, acting through the glucocorticoid receptor (GR), increases FoxO1 and reduces Sp1 levels. Together with cytostatic effects through targeting cell cycle inhibitors, we showed that FoxO1 represses VEGF mRNA levels. The downregulation of Sp1 will lead to a decrease in the levels of its transcriptional targets, which are known to include both VEGF and MMP-2. The reduction in VEGF will contribute to the lower activation of ERK1/2, Akt and RhoA GTPase, in turn influencing endothelial cell proliferation and migration. Corticosterone-mediated GR activation also may influence these proteins independently of the effects on FoxO1 and Sp1 (dotted lines). Akt exerts an inhibitory influence on FoxO1 protein (dashed line), thus the reduction in Akt activation will relieve this suppressive effect and promote higher levels of FoxO1 protein. Reduced production and activation of MMP-2 contributes to a decrease in proteolysis required for cell sprouting. The combined inhibitory effects on proliferation, migration and proteolysis effectively curtail the process of angiogenesis. Inhibitory influences are highlighted with red lines.

### Corticosterone Inhibits Endothelial Cell Migration and RhoA Activity

Using a scrape migration assay, we found that corticosterone significantly reduced endothelial cell migration distance (Control, 1033±142 µm vs. Corticosterone, 253±61 µm; p<0.0001) (**Figure 4A, B**). Cell movement requires coordinated activation of RhoGTPase family members, to allow for rearrangement of the actin cytoskeleton. We found that corticosterone pretreatment significantly downregulated RhoA activity (p = 0.008) (**Figure 4C**), but did not alter Rac1/2/3 or Cdc42 activities (p>0.05). Corresponding with the reduction in RhoA activity, we observed a reduction in actin stress fiber formation in corticosterone treated cells, (**Figure 4D**).

### Corticosterone Inhibits MMP-2 Production and Activation

MMP-2 is a key protease produced by endothelial cells [28] and is required for capillary sprouting [32]; thus, inhibition of either the production or activity of MMP-2 may contribute to reduced sprouting within the 3D collagen matrix. 48 hours of corticosterone treatment significantly reduced MMP-2 production and the percent of activated MMP-2 in the media conditioned by 3D cultured endothelial cells (**Figure 5A**). Similarly, treatment of monolayer endothelial cells with corticosterone for 48 hours reduced both total MMP-2 levels and percent of activated MMP-2 compared to control cells (**Figure S2A**). We have previously observed that changes in percent activated MMP-2 often reflect changes in expression or activity of MT1-MMP [28], [31]. Surprisingly, both whole cell (p = 0.006) and cell-surface localized MT1-MMP (p = 0.03) were significantly increased compared to control levels after 48 hours of corticosterone treatment (**Figure 5B**). Corticosterone treatment did not affect TIMP-2 production, but significantly reduced TIMP-2 secretion (p = 0.05) (**Figure 5C**).

The glucocorticoid receptor (GR) mediates transcriptional effects of glucocorticoids. We treated monolayer endothelial cells with the GR inhibitor RU 486 to determine if the direct transcriptional effects of GR signaling are required for corticosterone-mediated inhibition of MMP-2 production and activation. RU 486 treatment prevented the corticosterone-induced reduction in MMP-2 production (**Figure S2B**). RU 486 alone had a significant inhibitory effect on MMP-2 activation. There was no further inhibitory effect of corticosterone treatment on MMP-2 activation in the presence of RU 486.

Corticosterone treatment of endothelial cells decreased the mRNA levels of MMP-2 (p = 0.006) (**Figure 6A**). We found that transcriptional activity of the full length MMP-2 promoter was significantly decreased in corticosterone treated cells (**Figure 6B**). To identify a region in the MMP-2 promoter that is responsive to corticosterone, we tested a series of MMP-2 promoter truncations. The corticosterone mediated repression of transcription remained significant with the −1510 bp truncation, but was not significant at either the −1386 or −510 bp truncations.

### Transcriptional Regulators Mediating Effects of Corticosterone

While the GR may directly influence VEGF and MMP-2 transcription, we considered that glucocorticoid treatment also may exert regulatory effects on these pro-angiogenic molecules through modulating the levels of other transcription factors. FoxO1 has clearly defined roles in the inhibition of cell cycle and induction of apoptosis [33], and we found that FoxO1 mRNA (p = 0.04) and protein (p = 0.02) levels are elevated in endothelial cells treated with corticosterone for 48 hours (**Figure 7A, B**). Overexpression of constitutively active FoxO1 by adenoviral transduction resulted in decreased VEGF mRNA levels, however no changes were found in MMP-2 mRNA transcript levels (**Figure 7C, D**). GATA-2, c-jun and Sp1 have been shown to be involved in the transcriptional regulation of MMP-2 [29], [34], [35], as well as to modulate gene products involved in proliferation and migration [36], [37], [38]. Corticosterone treatment of endothelial cells did not modify GATA-2 or c-jun, but resulted in a significant decrease in Sp1 levels (p = 0.03).

## Discussion

In the current study, we demonstrate that a pathophysiological concentration of the endogenous glucocorticoid steroid corticosterone reduces the capillary number in rodent skeletal muscle and represses endothelial cell proliferation, migration and sprouting in culture. We found that corticosterone treated endothelial cells had lower VEGF mRNA levels, as well as reduced activation of downstream effectors of the VEGF signal pathway. MMP-2 production and activation was reduced in corticosterone treated cells, correlating with inhibition of cell sprouting within a 3D collagen matrix. Corticosterone increased the mRNA and protein levels of the cytostatic transcription factor FoxO1, and we demonstrated that FoxO1 modulates VEGF mRNA. Levels of Sp1, which is a known transcriptional regulator of both VEGF and MMP-2 [35], [37], also were reduced by corticosterone. These findings give further mechanistic insights as to how angiostatic steroids exert their inhibitory effects on endothelial cells **(** **Figure 8** **)**.

Chronic administration of corticosterone, the main endogenous glucocorticoid in rodents, resulted in significantly reduced number of capillaries per muscle fiber. This effect could have resulted from enhanced capillary regression or from repression of capillary sprouting. To further examine specific processes affected by corticosterone treatment, we utilized various *in vitro* culture models of endothelial cells. Endothelial cell monolayers (2D) treated with corticosterone did not appear phenotypically different from untreated cells, whereas in 3D cultures, endothelial cells became rounded. However, at the time point examined, the rounded cells remained viable, and did not show indications of increased apoptosis or necrosis. A 3D endothelial cell spheroid model in which sprouting originates from a central cell mass was used to quantify cell sprouting *in vitro*. Spheroids underwent extensive endothelial cell sprouting, with sprouting tips evident within several hours of plating in 3D collagen, and numerous extended sprouts visible after 24 hours. In contrast, the sprouts formed at 24 hours were largely destabilized and had regressed after 48 hours of corticosterone treatment. The dramatic morphological effects observed in 3D and spheroid cultures compared to monolayer cultured cells likely is a consequence of the dynamic changes in cell adhesion and motility that cells undergo within the 3D matrix, compared to the static and contact-inhibited status of 2D monolayers. The delayed effect of glucocorticoid treatment observed in the spheroid cultures may be a function of the longer time required for corticosterone to exert genomic effects [39], compared to the more rapid (and transcription-independent) initiation of tip cell sprouting. Similarly, it has been reported that glucocorticoid effects on endothelial cell intercellular junctions are detectable only after a prolonged treatment period [11], [40], [41]. Furthermore, considering that the relative potency of synthetic glucocorticoids can be as much as 25 times greater than that of endogenous cortisol/corticosterone [42], further investigation of the angiostatic effects of very low doses of synthetic glucocorticoids such as dexamethasone is warranted.

We found that glucocorticoid treatment decreased the mRNA levels of VEGF, as has been previously reported by others [8]. VEGF is a potent pro-angiogenic factor that promotes the activation of multiple signal pathways, including MAPK and Akt, leading to increased proliferation, migration and survival of endothelial cells [43]. The reduced phosphorylation of Akt and ERK1/2 in corticosterone treated cells thus is consistent with the decrease in VEGF production; however, these pivotal signaling intermediaries also may be influenced by corticosterone, independently of the VEGF pathway. ERK1/2 and Akt have well-described roles in regulating cell proliferation and motility [44], [45] and we previously showed that both ERK1/2 and Akt play critical roles in the process of microvascular network formation [34], [36]. Thus, the observed inhibition of endothelial cell proliferation in corticosterone treated cells may be the consequence of the reduction in phosphorylation of ERK1/2 and Akt. Functionally, corticosterone treated endothelial cells had decreased proliferative capacity.

Corticosterone treatment also substantially impaired endothelial cell migration, which is integral for the process of capillary sprout formation. We showed that corticosterone treatment resulted in downregulation of active RhoA in endothelial cells, consistent with a previous report that glucocorticoid treatment reduced levels of active RhoA in a blood-brain barrier culture model [46]. RhoA activity is associated with the formation of stress fibers, focal adhesions, and retraction of the trailing end of the cell [47]. Thus, downregulation of RhoA activity would have the effect of inhibiting endothelial cell migration as well as altering cell-matrix adhesion, which may underlie the phenotypic cell rounding observed in 3D cultured endothelial cells. VEGF is an activator of RhoA [48], [49], [50], thus the reduced production of VEGF in response to corticosterone also may contribute to the reduced RhoA activation.

Sprout formation within the interstitial matrix also requires the co-ordinated proteolysis of ECM components to enable invasion of the extending sprout. Our results show that corticosterone inhibits endothelial cell production and activation of MMP-2, an enzyme that is required for endothelial cell sprout formation [28], [51]. Elevated MMP expression (particularly MMP-9) in pro-inflammatory conditions has been controlled by use of pharmacological doses of dexamethasone (or alternative) treatment [52], [53], [54], [55]. It is notable, however, that we observed a significant reduction of MMP-2 at a substantially lower dose of corticosterone, indicating that pathophysiological levels of endogenous corticosterone can modulate MMP-2 production.

MT1-MMP is the main cell-surface activator of MMP-2, and as such is a critical regulator of MMP-2 activity [56]. While we expected to see a decrease in the levels of MT1-MMP with corticosterone treatment, we observed increases in MT1-MMP protein levels (both whole cell and membrane-localized). Interestingly, a similar effect is observed in cells following inhibition of PI3K/Akt [34], suggesting that a reduction in Akt signaling in the corticosterone treated cells may underlie this effect. Alternatively, glucocorticoids are known to insert into the plasma membrane and affect local membrane fluidity as well as interact with membrane proteins [57] and may thus interfere with MT1-MMP localization and function. We found that corticosterone decreased the secretion but not whole cell level of TIMP-2, suggesting impairment in the secretory process; however it is not clear whether this would influence the activation of MMP-2. Thus, the mechanism by which MMP-2 activation is repressed requires further investigation.

MMP-2 mRNA levels and promoter activity assays provided evidence that corticosterone represses the transcription of MMP-2. The MMP-2 promoter does not contain a canonical glucocorticoid responsive element (5′-GGTACAnnnTGTTCT-3′) [58]. However, analysis of the −1510 to −1386 bp region of the rat MMP-2 promoter revealed several predicted GR binding sites clustered at ∼−1400 bp (Signal Scan; http://www-bimas.cit.nih.gov/molbio/signal/). Alternatively, the effect of corticosterone on MMP-2 transcription may be mediated through other transcription factors. Goldman et al. [59] reported that the steroid progesterone mediates inhibition of MMP-2 transcription through enhanced degradation of the Sp family member Sp4, which results in reduced *trans*-activation of the MMP-2 promoter. Sp1 is an established *trans*-activator of the MMP-2 promoter [35]. We found that corticosterone significantly reduces Sp1 levels, which may contribute to the transcriptional repression of MMP-2 by corticosterone. Multiple predicted Sp1 binding sites are located within the −1510 to −1386 bp promoter region of MMP-2, consistent with our analysis of promoter truncations. Beyond its role as a regulator of MMP-2 transcription, Sp1 also is a major transcriptional regulator of VEGF-A [37], and thus, the reduction in Sp1 levels could underlie numerous effects of effects of corticosterone on endothelial cell migration, proliferation and survival.

FoxO1 mRNA and protein levels were increased substantially in corticosterone treated endothelial cells. FoxO1 is an established cell cycle repressor, through the combined actions of enhancing p27^Kip1^ transcription while reducing transcription of cyclinD1 [60]. Inhibition of FoxO1 has previously been shown to increase the migratory and sprout-forming capacity of endothelial cells [61]. In this study, we provide novel evidence that FoxO1 represses VEGF mRNA levels, but does not influence MMP-2 mRNA levels. Thus, FoxO1 may represent a critical effector of corticosterone-mediated effects on endothelial cell proliferation and migration.

This work helps to clarify how pathophysiological elevations in glucocorticoids, as is commonly observed in individuals with obesity, diabetes or Cushing’s syndrome, exerts dramatic angiostatic effects by regulating various processes that are crucial for angiogenesis. The combined repressive influences on proliferation, migration and proteolysis illustrate the efficacy with which corticosterone, and likely other glucocorticoids, inhibit angiogenesis and destabilize nascent capillaries. Defining the cellular effects of corticosterone on endothelial cells may help to develop manipulations that will reverse the pathological microvascular consequences of glucocorticoid dysregulation.

## Supporting Information

Figure S1 **Corticosterone does not cause apoptosis or necrosis of endothelial cells.** Skeletal muscle endothelial cell spheroids were suspended in a 3-dimensional type I collagen culture (A, B). Spheroids were treated for 48 hours with 600 nM corticosterone, prior to immunostaining for cleaved caspase-3 (red) and actin (green). Endothelial cells were either resuspended in 3D type 1 collagen (5.0×10^5^ cells/ml) or in monolayer cultures (1.0×10^6^ cells) plated in 35 mm^2^ dishes coated in type-1 collagen and treated with 600 nM corticosterone for 48 hours (C-E). Cells were stained with a live/dead cell quantification kit (Molecular Probes). Live cells (green) were detectable in all conditions, while minimal dead cells (red) were visible in any condition. C – Control, CORT – Corticosterone.
(TIF)

Figure S2 **Corticosterone inhibits MMP-2 production via glucocorticoid receptor activation.** Endothelial cells (1.0×10^6^ cells) were plated in 35 mm^2^ dishes coated with type I collagen and treated with 600 nM corticosterone for 24 and 48 hours. Total (Ai) and percent active (Aii) MMP-2 levels in whole cell lysates were measured by gelatin zymography (*p = 0.02 vs 48 hr control, n = 4; *p = 0.002 vs 48 hr control, n = 4, respectively) (A). Cells were also pre-treated with 10 µM RU 486 for two hours and then treated with corticosterone for 48 hours. Total (Bi) and percent activated (Bii) MMP-2 levels in whole cell lysates were measured by gelatin zymography (i: * p<0.01 vs control. ii) * p<0.01 vs control, # p<0.001 vs control, n = 6, respectively. C – Control, CORT – Corticosterone, RU - RU 486. N values represent independent experiments.
(TIF)
