# Supplementary figures and images for: Inhibition of Proliferation, Migration and Proteolysis Contribute to Corticosterone-Mediated Inhibition of Angiogenesis
Source: PLoS One. 2012 Oct 2;7(10):e46625. doi: 10.1371/journal.pone.0046625 (PMC3462789; doi:10.1371/journal.pone.0046625)

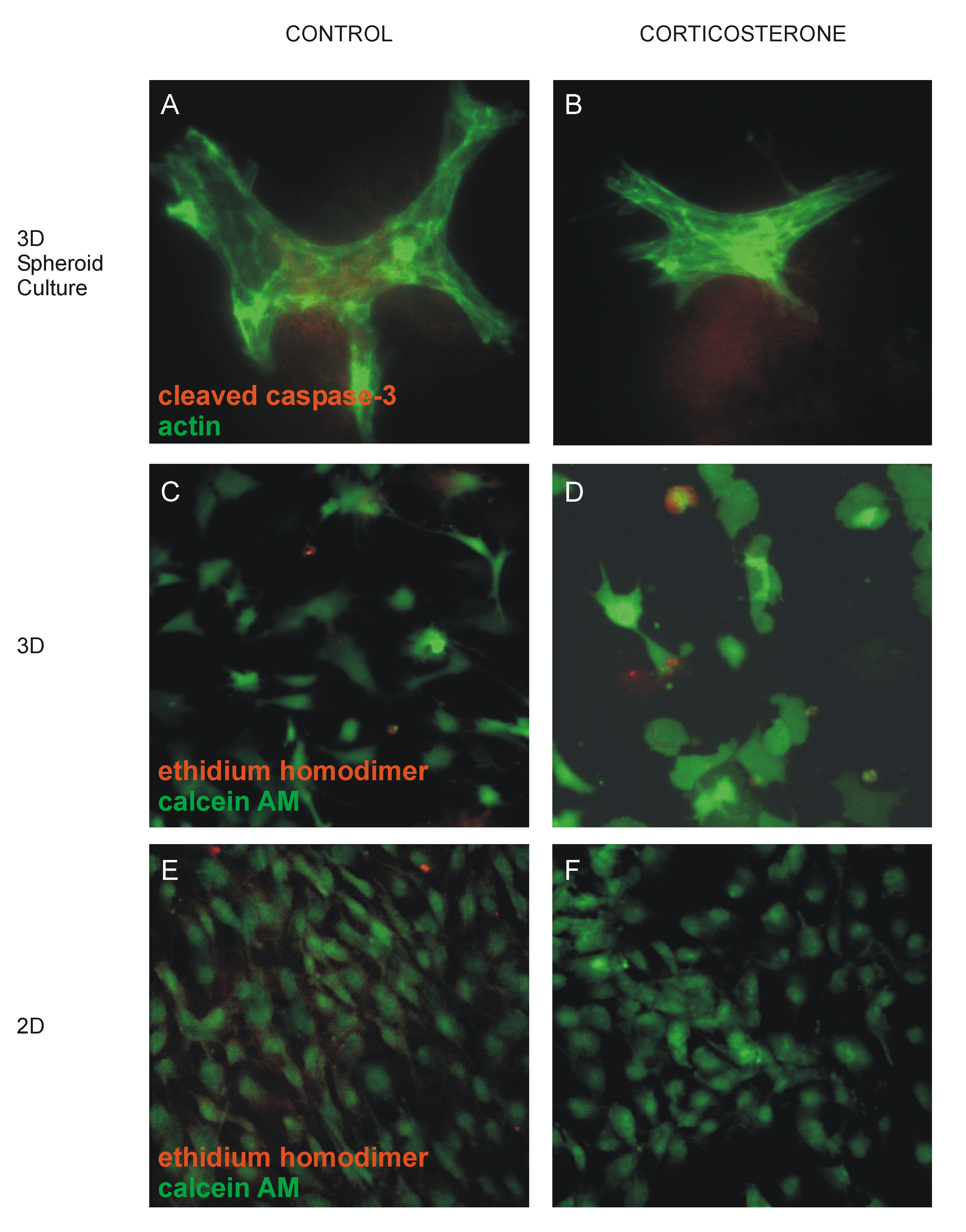

Supplement: Figure S1 — Corticosterone does not cause apoptosis or necrosis of endothelial cells. Skeletal muscle endothelial cell spheroids were suspended in a 3-dimensional type I collagen culture (A, B). Spheroids were treated for 48 hours with 600 nM corticosterone, prior to immunostaining for cleaved caspase-3 (red) and actin (green). Endothelial cells were either resuspended in 3D type 1 collagen (5.0×10^5^ cells/ml) or in monolayer cultures (1.0×10^6^ cells) plated in 35 mm2 dishes coated in type-1 collagen and treated with 600 nM corticosterone for 48 hours (C-E). Cells were stained with a live/dead cell quantification kit (Molecular Probes). Live cells (green) were detectable in all conditions, while minimal dead cells (red) were visible in any condition. C – Control, CORT – Corticosterone. (TIF) [file pone.0046625.s001.tif]

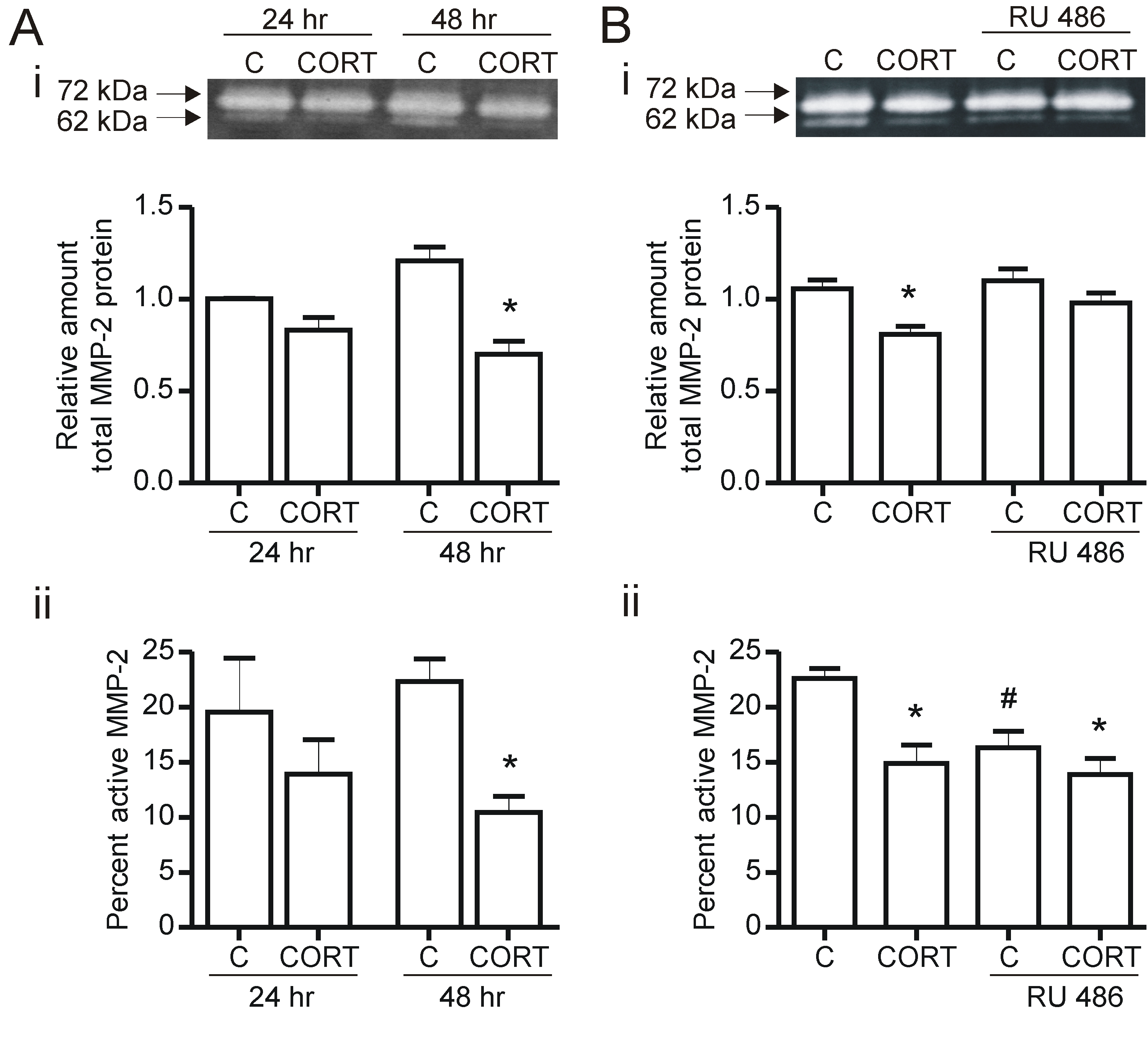

Supplement: Figure S2 — Corticosterone inhibits MMP-2 production via glucocorticoid receptor activation. Endothelial cells (1.0×10^6^ cells) were plated in 35 mm2 dishes coated with type I collagen and treated with 600 nM corticosterone for 24 and 48 hours. Total (Ai) and percent active (Aii) MMP-2 levels in whole cell lysates were measured by gelatin zymography (*p = 0.02 vs 48 hr control, n = 4; *p = 0.002 vs 48 hr control, n = 4, respectively) (A). Cells were also pre-treated with 10 µM RU 486 for two hours and then treated with corticosterone for 48 hours. Total (Bi) and percent activated (Bii) MMP-2 levels in whole cell lysates were measured by gelatin zymography (i: * p<0.01 vs control. ii) * p<0.01 vs control, # p<0.001 vs control, n = 6, respectively. C – Control, CORT – Corticosterone, RU - RU 486. N values represent independent experiments. (TIF) [file pone.0046625.s002.tif]
